# Lifestyle-Related Factors Associated with Reproductive Health in Couples Seeking Fertility Treatments: Results of A Pilot Study

**DOI:** 10.22074/ijfs.2018.5135

**Published:** 2018-01-15

**Authors:** Marie-Lou Piché Piché, Véronique Babineau, Julie Robitaille, Émilie Lachance, Stephanie-May Ruchat

**Affiliations:** 1 Department of Human Kinetics, Université du Québec à Trois-Rivières, Quebec, Canada; 2 Department of Obstetrics and Gynaecology, Centre intégré universitaire de santé et de services sociaux de la Mauricie-et-du-Centre-du-Québec, Affiliated to the University of Montreal, Trois-Rivières, Canada; 3 School of Nutrition, Laval University, Quebec, Canada

**Keywords:** Infertility, Lifestyle, Sleep Quality

## Abstract

**Background:**

The objective of this pilot study was to evaluate the feasibility of conducting a larger prospective cohort study, which will aim at determining the independent contribution of male and female lifestyle-related factors to assisted reproductive technology (ART) success. The study also examined whether couples seeking fertility treatments present lifestyle-related factors that may interfere with their reproductive health.

**Materials and Methods:**

This prospective pilot study was conducted in a fertility clinic between May 2015 and February 2016. Feasibility factors evaluated were recruitment rates, compliance with the protocol, retention rate and ART outcomes at six-month follow-up. Anthropometric profile and lifestyle habits of both partners were evaluated before the beginning of infertility treatments.

**Results:**

We approached 130 eligible infertile couples. Among them, 32 (25%) agreed to participate and 28 (88%) complied with the protocol. At six-month follow-up, seven couples (25%) did not start, or stop, infertility treatments and 13 couples (62%) achieved a clinical pregnancy. Among the 28 couples included in the analyses, 16% of the partners were obese and 23% had abdominal obesity. The majority of the subjects were still drinking alcohol (84%). Sixty-eight percent of women needed improvement in their diet (vs. 95% of men, P=0.05) and none of them achieved the Canadian recommendations for physical activity (vs. 33% of men, P=0.001). Moreover, 35% of the partners had a poor sleep quality. Overall, women presented a worse reproductive health profile than men, with 3.1 and 2.4 out of seven adverse factors, respectively (P=0.04).

**Conclusion:**

Conducting a large prospective cohort study in our fertility clinic will be feasible but recruitment and compliance with the protocol need to be improved. Many women and men seeking fertility treatments present unfavourable lifestyle-related factors that may explain, at least partially, their difficulties in conceiving.

## Introduction

It is well recognized that various lifestyle-related factors, such as obesity, smoking, other substance abuse and heavy alcohol consumption, have a negative impact on both male and female fertility, and the success of assisted reproductive technology (ART) (1, 2). Other lifestyle habits, such as mild-to-moderate alcohol consumption, caffeine intake, nutritional factors or exercise may also negatively affect reproductive health; however, the available evidence is inconclusive (2).

Obesity is associated not only with female infertility (3, 4) but also with decreased implantation and live birth rate after ART (1, 2). In men, obesity has been linked to an increased prevalence of azoospermia or oligozoospermia (5), a reduced ejaculate volume (6) and a higher risk of sperm DNA damage (7). Similarly, tobacco smoking and ductive health. In females, smoking is associated with an increased risk of infertility and lower success rate from ART (1, 2) whereas alcohol consumption has been linked to hormonal and menstrual dysfunction, and has a negative impact on embryo implantation (1, 2). In men, smoking is associated with impaired semen quality (8) and alcohol consumption contributes to testicular atrophy, reduced libido and alterations in semen parameters (1, 2). Finally, evidence also suggests that caffeine intake has a potential dose-response association with a longer time to conception (1). Other lifestyle habits, such as physical activity levels (9-11), nutritional factors (12-15) and sleep quality (16) might also negatively affect female and/or male fertility and ART outcome. 

The above-mentioned literature therefore suggests that infertile people can take non-medical actions, such as maintaining healthy body weight and lifestyle habits to improve their chance of conception, spontaneously or following ART. However, studies have shown that many women undergoing fertility treatments tend to make poor lifestyle choices that may affect their chance of conception. A significant proportion of these women continue to drink caffeine and alcohol (10, 17, 18) and do not make lifestyle changes to improve their chances of becoming pregnant (18). Importantly, the majority of the studies that evaluated lifestyle-related factors in fertility clinic settings, were conducted in women. Fewer studies were conducted in men and to the best of our knowledge, only one pilot study including 23 infertile couples documented lifestyle-related factors in both partners (19). Evaluating lifestyle-related factors in both partners and identifying those contributing to ART success, are essential to develop targeted recommendations to help infertile couples to conceive a child.

In this pilot study, we evaluated the feasibility of conducting a larger prospective cohort study that will aim at determining the independent contribution of male and female lifestyle-related factors to ART success. The study also examined whether couples seeking fertility treatments present unfavorable lifestyle-related factors that may interfere with their reproductive health and evaluated possible differences in these factors between men and women.

## Materials and Methods

Heterosexual couples seeking fertility treatments for the first time and being able to understand, speak and write French were eligible to participate in this prospective pilot study. Recruitment took place at the fertility clinic of the Centre hospitalier affilié universitaire régional (CHAUR) de Trois-Rivières (Qc, Canada) between May 2015 and February 2016. Men and women who agreed to participate in our study were assessed prior to the initiation of infertility treatments. The couples were followed- up for six months to assess ART success, defined as the confirmation of a clinical pregnancy. This project was approved by the Centre intégré universitaire de santé et de services sociaux de la Mauricie et Centre-du-Québec (CIUSSS MCQ) and the Université du Québec à Trois- Rivières Ethics Committes. Written informed consent was obtained from all couples participating in the study.

## Assessment of feasibility

To assess the feasibility of a larger prospective cohort study, recruitment rates, compliance with the protocol (defined as fulfilling the questionnaires and wearing the accelerometer as requested), as well as retention rate and ART outcomes at six-month follow-up were evaluated.

## Assessment of anthropometric profile

Height was measured to the nearest millimetre using a standardized cloth tape measure, and body weight was measured to the nearest 0.1 kg on a calibrated balance after removing shoes (UM016 2202, Tanita Corporation, USA). Body mass index (BMI) was then calculated in kilograms per meter squared (kg/m^2^). On the basis of international BMI cut-off values for adults, the prevalence of underweight (<18.5 kg/m^2^), normal weight (18.5-24.9 kg/m^2^), overweight (25.0-29.9 kg/m^2^) and obese (=30.0 kg/m^2^) were calculated. Waist circumference (WC) was measured using a standardized cloth tape measure according to standard procedures (20). Abdominal obesity was defined as WC=102 cm in men and =88 cm in women (21).

## Assessment of lifestyle habits

Each partner received an e-mail containing instructions for completing online questionnaires assessing their eating and sleeping habits. A web-based self-administered food frequency questionnaire (web-FFQ), containing a list of typical foods available in the province of Quebec, was used to assess dietary intakes over the last month. The test-retest method showed that this questionnaire has good reliability (mean R=0.72, 95% confidence interval 0.68; 0.76) (22). From the data collected by the web-FFQ, we calculated a diet quality index based on Kennedy’s healthy eating index (HEI), adjusted to Canadian recommendations. The Kennedy’s healthy eating index includes 10 components (grain products, vegetables and fruits, meat and alternatives, milk and alternatives, total fat, total saturated fatty acids, cholesterol, sodium and variety). A maximum of 100 points is possible, which would correspond to a perfect diet. We categorized partners as having a good diet (>80 points), a diet that needs improvement (50-80 points) and a poor diet (<50 points) (23). The web-FFQ also allowed assessing alcohol and caffeine consumption. Adverse behaviors related to reproductive health were defined as consuming more than two caffeinated drinks per day (>200 mg/day of caffeine) for women (1, 24) and consuming any alcohol for men and women (1, 2). Studies evaluating the relation between caffeine consumption and men reproductive health are limited, and therefore, no recommendations are available for men trying to conceive.

The pittsburgh sleep quality index (PSQI) was used to assess sleep quality over a month. PSQI consists of 19 items, each weighted on a 0-3 interval scale, generating seven “component” scores. The final score can vary from a minimum of 0 (no sleeping difficulty) to a maximum of 21 (significant sleeping difficulty). A score ≤5 is associated with good sleep quality, whereas a score >5 is associated with poor sleep quality (25). 

To objectively assess current physical activity levels of the partners, we asked them to wear an accelerometer over their hip on an elastic belt from wake-up time to bedtime, for seven consecutive days. The participants were asked to remove the accelerometer when sleeping, showering or performing water activities. Furthermore, they received a daily diary to document wear and non-wear time periods. We used the triaxial ActiGraph GT3X accelerometers (ActiGraph, Pensacola, FL). The ActiGraph GT3X measures data in a 60-s epoch and has been widely used in research for assessing physical activities in adults. The accelerometer provides measures such as activity intensity and duration, step counts, and energy expenditure and has been shown to reasonably correlate with doubly labeled water-derived, the gold standard to assess energy expenditure (26). Valid data were defined as four days of monitoring for 10 hours of wear time per day (27). Participants were asked to maintain their usual activities. Data were processed using the Actilife software version 6.13.2 (ActiGraph, LLC, FL, USA). The accelerometer data obtained were averaged across valid wear days. To derive the activity frequency, intensity and duration from the measured activity in counts per minute per day, the Freedson equation was used: sedentary (<100 counts), light (100-1951 counts), moderate (1952-5724), vigorous (5725-9498), and very vigorous (>9498) (28). Non-wear time was defined as previously suggested (27).

A global “reproductive health score” was calculated by attributing 1 point per adverse factor related to women and men reproductive health (for women: age=35 years old, BMI =30 kg/m^2^, waist circumference =88 cm, consuming alcohol (=1 unit/week), HEI<50, <150 minutes of moderate to vigorous physical activity (MVPA) in bouts of =10 minutes, poor sleep quality (score>5); for men: age=45 years old, BMI=30 kg/m^2^, waist circumference =102 cm, consuming alcohol (=1 unit/week), HEI<50, <150 minutes of MVPA in bouts of =10 minutes, poor sleep quality (score>5).

Data on sociodemographic status, reproductive history, smoking and drug use, personal and family medical history, as well as causes of infertility, infertility treatments received and biochemical and clinical pregnancy were gathered from patients’ medical records.

### Statistical analysis

Means and standard deviations, as well as percentages, were computed for men and women for socio-demographic and anthropometric characteristics. The normality assumption was tested using the Shapiro-Wilk test. Because several variables were not normally distributed and our sample size was small, we used the Wilcoxon-Mann-Whitney nonparametric test to compare lifestyle-related factors between men and women. For categorical variables, we used the Fisher's exact test. Statistical analyses were performed by using SPSS statistical software (version 23.0) and results were considered to be significant at P≤0.05).

## Results

### Feasibility

**Fig.1:**
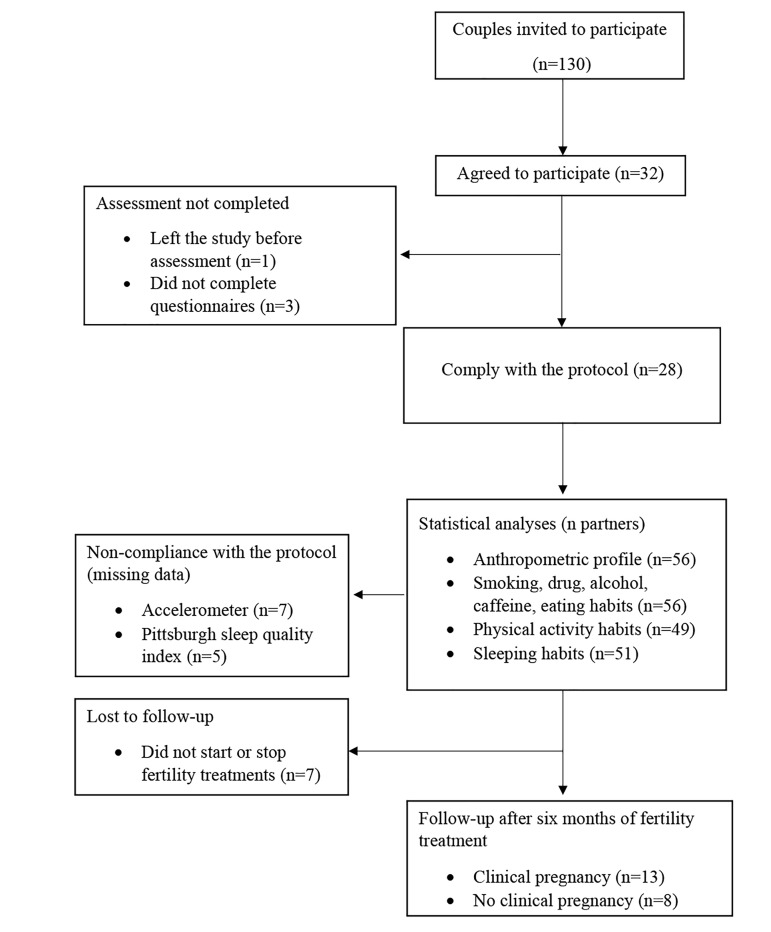
Flow diagram of recruitment, compliance with the protocol and retention of the study population.

Between May 2015 and February 2016, 130 eligible couples were approached and asked whether they were interested in participating in our pilot study. Thirty-two couples agreed to participate (25% recruitment rate). Reasons for not agreeing to participate were: not interested in the study, lack of time or overwhelmed by medical exams and treatments for infertility. Among the 32 couples, one left the study before having completed the questionnaires and worn the accelerometer. Three couples were excluded from the analyses because of non-compliance with fulfilling questionnaires by the man, leaving 28 couples for the analyses (Fig .1). 

These 28 couples had missing data in the data set, especially for objective physical activity measures. Seven participants (12.5%) did not wear the accelerometer for at least four days of monitoring for 10 hours of wear time per day. Incomplete PSQI (n=5, 9%) was also another source of missing data. Seven couples (25%) did not start, or stopped, infertility treatments at six-month follow-up. Thirteen couples (13 out of 21, 62%) achieved a clinical pregnancy whereas 8 couples (8 out of 21, 38%) did not.

### Characteristics and lifestyle-related factors of couples seeking fertility treatments

A description of the socio-demographic characteristics of the 28 couples included in the analyses, is provided in Table 1. According to this table, in general, the partners were in their thirties, were well educated and did not have a child. The cause of infertility of the couple was of female origin in 46.4% of the cases, of male origin in 17.9% of the cases, of male and female origin in 14.3% of the cases, and of unknown reasons for 21.4% of the cases.

**Table 1:** Socio-demographic characteristics of couples who underwent infertility treatments


Variable	Women n=28	Men n=28

Age (Y)	32.0 ± 4.4	35.6 ± 8.4
	(25.0-42.0)	(25.0-58.0)
Women≥35 years old	10 (36)	-
Men≥45 years old	-	5 (18)
Maternity/Paternity		
No	22 (78)	19 (68)
Yes, with actual partner	3 (11)	2 (7)
Yes, with ex-partner	3 (11)	6 (21)
Yes, with actual and ex-partner	0 (0)	1 (4)
Educational level		
No-university degree	13 (46)	11 (39)
University degree	15 (54)	17 (61)
Cause of infertility	
Female	13 (46.4)	
Male	5 (17.9)	
Female and male	4 (14.3)	
Unknown	6 (21.4)	


Data are presented as means ± SD (minimum-maximum) or n (%).

Anthropometric profile and lifestyle habits related to reproductive health of the 28 couples (56 individuals) are presented in Table 2. Overall, 16% of them were obese and 23% had abdominal obesity. Only three individuals were smokers (one woman and two men); the two men who smoked tobacco also reported smoking marijuana on a weekly basis. Most partners (84%) were still drinking alcohol (≥ 1 drinks per week). No statistical difference in these lifestyle- related factors were found between men and women. Twenty-one percent of women were consuming more than the recommended 2 cups of caffeinated drinks per day. Eating habits were worse in men than in women, with 95% of them having a poor diet quality or a diet quality needing improvement (versus 68% of women, P=0.05). On the other hand, physical activity habits were better in men, with 33% of them achieving the Canadian recommendations for physical activity (versus 0% of women, P=0.001). A poor sleep quality was present in 35% of the partners with no difference between men and women.

When considering the seven lifestyle-related factors associated with reproductive health (age, BMI, WC, alcohol, diet, physical activity and sleep) in men and women for which all the data were available (n=44), we found that 9% of men and 41% of women presented at least four adverse factors (P=0.08), with a mean of 3.1 and 2.4 adverse factors observed in women and men, respectively (P=0.04, Table 3).

**Table 2:** Lifestyle-related factors associated with unfavorable reproductive health of couples about to undergo fertility treatments


Variable	All	Women	Men	P value

Anthropometric profile	n=56	n=28	n=28	
BMI (kg/m^2^)	25.7 ± 4.9	29.9 ± 5.5	26.6 ± 4.3	0.08
UW	2 (3.6)	2 (7.1)	0 (0)	
NW	25 (44.6)	15 (53.6)	10 (35.7)	0.13
OW	20 (35.7)	6 (21.4)	14 (50)	
OB	9 (16.1)	5 (17.9)	4 (14.3)	
Abdominal obesity^a^	13 (23.2)	9 (32.2)	4 (14.3)	0.10
Smoking	n=56	n=28	n=28	
Yes	3 (5.4)	1 (3.6)	2 (7.1)	0.49
Drug use	n=56	n=28	n=28	
Yes	2 (3.6)	0 (0%)	2 (7.1)	0.15
Drinking/Eating habits	n=56	n=28	n=28	
Alcohol (unit/week)	6.1 ± 6.7	4.3 ± 3.7	7.9 ± 8.5	0.05
≥1 unit/week	47 (84)	23 (82.2)	24 (85.7)	0.57
Caffeine (mg/day)	153.8 ± 144.7	112.8 ± 88.0	194.8 ± 177.2	0.11
>200 mg/day*	-	6 (21.4)	-	
Diet quality index	69.2 ± 11.8	72.0 ± 12.4	66.4 ± 10.8	0.10
Good diet	11 (19.6)	9 (32.1)	2 (7.1)	0.05
Diet needing improvement	41 (73.2)	18 (64.3)	23 (82.2)	
Poor diet	4 (7.2)	1 (3.6)	3 (10.7)	
Physical activity habits	n=49	n=25	n=24	
Time spent at MVPA (minutes/day)	34.2 ± 38.8	24.3 ± 11.8	44.5 ± 52.8	0.05
Not achieving≥150 minutes of MVPA per week	16 (32.7)	10 (40)	6 (25)	0.36
Time spent at MVPA in bouts≥10 minutes (minutes/day)	13.5 ± 23.5	7.9 ± 6.3	19.3 ± 32.2	0.46
Not achieving ≥150 minutes of MVPA in bouts of ≥10 minutes	41 (83.7)	25 (100)	16 (66.7)	0.001
Time spent in sedentary activity (hours/day)	9.1 ± 1.7	9.2 ± 1.3	9.0 ±1.9	0.50
Sleeping habits	n=51	n=25	n=26	
Sleeping score	5.2 ± 2.7	5.16 ± 3.2	5.23 ± 2.3	0.49
Overall poor sleep quality	18 (35.3)	7 (28)	11 (42.3)	0.38


Data are presented as mean ± SD or n (%). P values indicate differences between women and men. BMI; Body mass index, MVPA; Moderate-to-vigorous intensity physical activity, OB; Obese, OW; Overweight, UW; Underweight, NW; Normal weight, ^a^; Abdominal obesity was defined as: waist circumference>88 cm in women, >102 cm in men, and *; No recommendations regarding caffeine intake are available for men trying to conceive.

**Table 3:** Overall number of adverse factors related to women and men reproductive health


	Overall n=44	Women n=22	Men n=22	P value

Number of factors^a^				
0	0	0	0	0.08
1	5 (11.4)	1 (4.5)	3 (13.6)	
2	15 (34.1)	7 (31.8)	9 (41.0)	
3	13 (29.5)	5 (22.7)	8 (36.4)	
≥4	11 (25.0)	9 (41.0)	2 (9.0)	
Global score^b^	2.8	3.1	2.4	0.04


BMI; Body mass index, MVPA; Moderate to vigorous intensity physical activity, HEI; Health eating index, ^a^; Among the following factors: for women: age ≥35 years old, BMI ≥30 kg/m^2^, waist circumference>88 cm, consuming alcohol (≥1 unit/week), HEI<50, <150 minutes/week of MVPA in bouts of ≥10 minutes, poor sleep quality (score>5). For men: age ≥45 years old, BMI ≥30 kg/m^2^, waist circumference >102 cm, consuming alcohol (≥1 unit/week), HEI<50, <150 minutes/week of MVPA in bouts of ≥10 minutes, poor sleep quality (score>5), and ^b^; The global score was calculated by attributing 1 point per adverse factor related to women and men reproductive health.

## Discussion

This pilot study demonstrated the feasibility of conducting a large prospective cohort study at the fertility clinic of the CHAUR of Trois-Rivières but also highlighted the need for improvement of several aspects of the protocol. First, recruitment rate was 25%. It is not possible to compare our recruitment rate with other similar studies because studies evaluating lifestyle-related factors in both partners, using detailed questionnaires and accelerometers, are inexistent. Nevertheless, recruitment was challenging and different explanations may be given. Men and women were recruited at the same time. Several men declined to participate in our study, which prevented us to recruit the couple. Working in close relationship with the medical team and delivering persuasive message to raise men interest in our study, will be essential to improve recruitment rate. In addition, the couple received a large amount of complex information about the medications, tests and procedures involved in infertility treatments on the day we invited them to participate in our study. They may have been overwhelmed and less inclined to participate in our study. Therefore, a better moment to approach the couples should be considered. Finally, the accelerometer to wear during seven days may have discouraged some couples to participate in our study.

Second, missing data were apparent in the data set in terms of sleeping and physical activity data. It will be essential to emphasize the importance of following the instructions provided in the questionnaires on how to respond to questions as well as wearing the accelerometer for at least 10 hours per day for four days in order to avoid missing data. Finally, at six-month follow-up, seven couples (25%) did not start, or stopped, infertility treatments for medical or personal reasons. This attrition rate will have to be taken into account when designing our larger prospective cohort study.

Our preliminary results also showed that many couples seeking infertility treatments present unfavourable lifestyle- related factors that may explain, at least partially, their difficulty in conceiving and affect future infertility treatment outcomes. Importantly, 41% of women and 9% of men presented at least four adverse factors that may have a negative impact on reproductive health. More specifically, 18% of women and 14% of men were obese, proportions similar to those reported by previous studies conducted in infertile populations (9, 11, 18, 29). In Canada, the prevalence of obesity in adults of reproductive age is slightly lower (22.4% in men and 16.6% in women).

Obesity is a well-known risk factor for female infertility, as it is related to ovulation and hormonal disorders (3, 4). In women who achieved a pregnancy, failure to achieve a live birth increased with higher BMI. In men, obesity may also affect fertility by altering sperm parameters (30). The localization of fat mass may also be important with regards to reproductive health. Abdominal obesity is associated with metabolic disorders, including insulin resistance, which may exert effects upon the hypothalamic- pituitary-ovarian (HPO) axis. Disturbances of the HPO axis may lead to alterations in sex hormone secretion and/ or metabolism, which may in turn cause hyperandrogenism and polycystic ovaries syndrome (PCOS) in obese women and hypotestosteronemia in obese men.

Despite the recommendation that people trying to conceive should not drink alcohol (1), we found that 82% of women and 86% of men were still drinking alcohol at the time they were seeking infertility treatments. Data from the 2012 Canadian Alcohol and Drug Use Monitoring Survey showed that 65.8% of adults (>25 years old) had drunk alcohol in the past month, which is lower than what we observed in our sample. Other studies also reported excessive alcohol intake by a significant number of women (any alcohol, 49%-73%) and men (>2 drinks/ day, 17%) undergoing infertility treatments (17-19, 29). It has also been recommended to women who are trying to conceive to reduce their caffeine intake (=200 mg per day) (1). In our study, 21% of the women consumed caffeine more than the recommended amounts, which is less than the previously reported rate (35-50%) (17, 29). In Canada, women of reproductive age (19-50 years old) consume 265 mg of caffeine per day (31), on average. This suggests that women of our study who are trying to conceive, slightly decrease their caffeine intake. Recommendations regarding caffeine intake in men trying to conceive are not available because the potential adverse effects of caffeine on male reproductive function have not been investigated extensively.

Finally, other lifestyle habits, such as nutrition, physical activity and sleeping habits may have a negative impact on fertility and ART outcome, but the currently available evidence is inconclusive. Ruder et al. (15) have reported that antioxidant intake was associated with shorter time to pregnancy, but this association varied according to BMI and age. A case-control study (n=61) found that fruit and vegetable intake could maintain or improve semen quality (14). A cohort study, conducted in men undergoing intracytoplasmic sperm injection cycles, has reported that semen parameters were negatively affected by the consumption of alcohol and red meat, but positively influenced by fruits and cereals consumption. The consumption of red meat also had a negative impact on fertilization and implantation rate (12). To the best of our knowledge, our study is the first to assess overall diet quality in infertile couples. We calculated a diet quality index based on Kennedy’s healthy eating index (23) and found that although the number of women who had a good diet quality was higher than men, the majority of the partners needed to improve their eating habits.

Physical activity has been associated with improved ART outcome. Evenson et al. (9) have reported that women who have been active in the year preceding infertility treatments, were more likely to have favorable pregnancy outcome, whereas two studies have shown that women who remained active during infertility treatments had higher implantation and live birth rates (10, 32). Importantly, these three studies did not assess physical activity objectively. Only one study measured physical activity in infertile men using accelerometers and found an inverted U-shape association between the number of bouts of MVPA and semen quality (11). These findings suggest that too little or too much physical activity may be detrimental for male reproductive health. Current physical activity recommendations for adults are to accumulate 150 minutes per week of MVPA in bouts of =10 minutes (33). Although no specific recommendations are available for people trying to conceive, it is reasonable to think that these recommendations are also valid for them. Only 16% of our subjects were meeting these recommendations, which is similar to the data from the Canadian Community Health Measure Survey showing that 16% of adults of childbearing age reached these recommendations using accelerometer-derived data (27).

Finally, sleeping habits is increasingly recognized as an important factor of human health and well-being (34). However, the relationship between sleep quality and reproductive health is largely unknown. Possibly, sleep disturbances are related to high levels of stress and anxiety symptoms, which may be associated with fertility problems. Sleep may therefore indirectly affect reproductive health (16). In our study, we found that 28% of women and 42% of men had poor sleep quality. A previous study examining sleep quality in infertile women using the PSQI, reported that 35% of them had disturbed sleep (35). While there is evidence suggesting that sleep disturbances may affect testosterone production and semen parameters (16), no studies have examined sleep quality in infertile men.

While our data are interesting and appear to be feasible to collect, they should be considered preliminary and descriptive. The small sample size should be acknowledged, yet the primary objective of this pilot study was to evaluate the feasibility of a prospective cohort study. Consequently, we did not have the power to detect differences in lifestyle-related factors associated with reproductive health between men and women. Similarly, we did not have the power to compare baseline lifestyle-related factors between couples who achieved a clinical pregnancy and those who did not. Another limitation of our study is that the population was homogenous with respect to race/ ethnicity and educational level, with the majority of recruited couples being highly educated.

We do not know whether lifestyle-related factors of the couples who agreed to participate were any different from those who did not agree to participate. The detailed questionnaires about eating and sleeping habits, as well as the accelerometer to wear during seven days, may have attracted more motivated and healthier couples. But, still, we observed a high proportion of unhealthy anthropometric profile and lifestyle habits despite having well- educated participants. These different factors suggest that a recruitment bias is likely to be present in our larger prospective cohort study, limiting the generalizability of our results to a wider population of infertile couples. Finally, although accelerometers provide a valid and objective measure of physical activity levels, non-waterproof accelerometers underestimate several type of physical activity, such as water activities. It is therefore possible that we underestimated the level of physical activity for some participants who removed the accelerometer to do water activities but the underestimation would be minimal. Only 6 participants (11%) of our subjects reported doing water activities; however, data were considered invalid for three of them because the accelerometer was worn for less than 10 hours. The three other participants reported only one hour of water activities during the wearing period.

The literature shows that a number of lifestyle-related factors have unfavourable effects on reproductive success of infertile men and women; however, further prospective cohort studies assessing both partners’ lifestyle-related factors, especially nutrition, physical activity and sleeping habits, will be needed to fully understand the independent contribution of male and female factors to ART success. Such large prospective cohort studies are essential to develop targeted recommendations to help infertile couples to conceive a child and this pilot study will help us to design such a prospective cohort study. 

## Conclusion

Though this pilot study had limitations, it provides us with key information that will help us to design a large prospective cohort study. Especially, improvement of recruitment strategies and directives to increase the compliance with the protocol will be essential to ensure its success. It also shows that a considerable proportion of men and women seeking infertility treatments present with several unfavourable lifestyle-related factors that may interfere not only with their fertility but also with future infertility treatment outcome. Conducting a large prospective cohort study will allow us to identify the independent contribution of male and female lifestyle-related factors to ART success. Such a study is essential to help designing interventions aimed at helping infertile couple to conceive a child. 
